# Celebrity CEOs and firm innovation investment: Evidence from Chinese-listed companies

**DOI:** 10.3389/fpsyg.2022.978946

**Published:** 2022-11-21

**Authors:** Dong Shao, Kangyin Lv, Shukuan Zhao, Shuang Wang

**Affiliations:** ^1^Business School, Northeast Normal University, Changchun, China; ^2^School of Management, Jilin University, Changchun, China

**Keywords:** celebrity CEOs, innovation investment, analyst coverage, SOEs, Chinese context

## Abstract

In today’s commercial-oriented world, intense social attention makes it easier for CEOs to become celebrities. This social escalation and characteristic change of CEOs into celebrities tend to influence their motivation and behavior, and thus the strategic decisions and results of firms. Despite the significance of recognizing CEOs’ social identity, the impact of celebrity CEOs on innovation strategy remains unknown. Integrating identity and upper echelons theories, this study examines and provides empirical evidence on how celebrity CEOs affect firm innovation investment using data of Chinese listed companies from 2015 to 2020. We argue that celebrity CEOs’ engagement in innovation investment is driven by their motivation for preserving celebrity status. Further, we show that analyst coverage plays a positive moderating role between celebrity CEOs and innovation investment, and the positive effect of celebrity CEOs on innovation investment becomes weaker in state-owned enterprises. This study confirms the important role of CEOs’ specific social identity in firm innovation strategy, which is motivated by celebrity CEOs’ attempt to maintain their established status and reputation. The results expand the research on the influencing factors of firm innovation investment that focus on executives’ social characteristics. They also provide managerial implications for board of directors to recruit and supervise a celebrity CEO.

## Introduction

Traditionally, a celebrity is a social actor who is more likely to work in the entertainment industry, receives widespread and public attention, and has profit-generating value (Treadway et al., 2009). However, recently, the business community has also captured the public’s attention, particularly, executives who lead successful companies and achieve great firm performance (Lovelace et al., 2018; Kim and Lee, 2022). Such executives are conferred with various top awards by social media, thus obtain the celebrity status and become celebrities (Hayward et al., 2004; Wade et al., 2006). The growing popularity of both Internet technology and social media has provided the public with more information channels and faster speeds for information transmission to know celebrity executives. Consequently, some outstanding CEOs have become household names at both local and global levels (Lovelace et al., 2018). Using their celebrity status as a form of enhanced social position legitimized and reinforced by the media and public opinion, such celebrity CEOs have garnered widespread attention and developed positive image (Rindova et al., 2006; Lee et al., 2020).

Celebrity CEOs have enormous and continuous influence on their firms (Treadway et al., 2009). Early research on celebrity CEOs examined their roles and the impact of their celebrity status on firm and individual interests. Celebrity CEOs bring intangible assets to their firms, such as signaling improved development prospects, increasing investor confidence, attracting extra resources, and promoting stock prices (Rindova et al., 2006). Such valuable intangible assets and CEOs’ ability to manage them create more value for organizations (Vatamanescu et al., 2022). Meanwhile, they receive benefits such as higher compensation, increased stock options, additional board seats in other firms, and better job opportunities (Hayward et al., 2004; Wade et al., 2006). Recently, some scholars studied the impact of celebrity CEOs on organizational behaviors and outcomes, such as acquisition premiums, corporate social responsibility, managerial risk-taking, and firm performance (Cho et al., 2016; Shi et al., 2017; Wei, 2021). However, little research has been undertaken to investigate the influence of celebrity CEOs on firms’ innovation activities.

Innovation is crucial to ensuring the survival of a company and promoting its development (Balkin et al., 2000). In particular, R&D expenditures have been shown to improve firm performance (Camison and Villar-Lopez, 2014; Hatzikian, 2015). Thus, improving the innovation capabilities of enterprises has attracted considerable attention from both researchers and practitioners. The upper echelons theory argues that firm behavior is an expression to the values and cognitive abilities of top managers (Hambrick and Mason, 1984). As one of the most important decision makers in a firm, the CEO’s cognitive pattern and value orientation, shaped by personal background and life experience, can influence strategic decisions (Li, 2013). Particularly, top managers’ characteristics have impact on firms’ innovation activities, such as technical background, education, tenure, age, and career horizon (Hambrick and Mason, 1984; Hambrick, 2007; Lin et al., 2011; Heyden et al., 2017). However, few studies have investigated the effect of CEOs’ celebrity status on innovation strategy and the psychological mechanisms underlying this process.

Based on upper echelons and identity theories, this study addresses the gap in the existing literature by investigating the relationship between celebrity CEOs and firm innovation, while considering the factors of analyst coverage and nature of firm ownership. Based on the panel data of Chinese firms listed on the Shanghai and Shenzhen stock exchanges from 2015 to 2020, we hypothesize that celebrity CEOs tend to increase innovation investment, as an effective method to promote firm performance, to maintain their public image as successful and visionary business leaders. Further, we postulate that when celebrity CEOs perceive higher expectations from either the public or themselves regarding their identity, they will have an increased motivation to engage in innovation activities. Hence, analyst coverage could be a key factor in promoting firm innovation investment through raising the celebrity CEOs’ perceived expectations. Additionally, we argue that firms’ ownership nature could also influence the relationship between celebrity CEOs and innovation investment, since the innovation willingness and motivation of executives are different between state-owned enterprises (SOEs) and non-SOEs.

This study makes the following contributions to the current literature. First, by introducing identity theory into the research framework of upper echelon theory, it analyzes the mechanism through which celebrity CEOs affect firm innovation investment from a psychological perspective. It expands the research on post-economic effects of CEOs’ celebrity status and provides unique insights into executives’ behavioral motivation in different social status. Second, we clarify the relationship between executives’ social characteristics and firms’ innovation strategy. The results contribute to the research field of innovation management by exploring the antecedents of innovation input, revealing executives’ social status as a decision-making reference for firms’ innovation strategy. Moreover, it provides unique insights into principle-agency conflict from the perspective of social expectations and executives’ self-supervision. CEOs’ celebrity status create an additional and informal governance mechanism that disciplines executives’ behaviors through their motivation to maintain their celebrity status, making stakeholders’ long-term interests consistent with the CEOs’ personal interests.

## Theoretical model and hypotheses development

### Celebrity CEO and firm innovation investment

According to upper echelons theory, executives make strategic decisions with partially personalized perspective that is derived from their individual experiences and characteristics (Hambrick and Mason, 1984; Hambrick, 2007). Scholars have explored the effect of CEO awards on their organizations (Wade et al., 2006; Graffin et al., 2008; Malmendier and Tate, 2009). For instance, corporate social responsibility (CSR) is heightened by the presence of celebrity CEOs (Lee et al., 2020). Moreover, companies with such CEOs tend to pay smaller premiums in mergers and acquisitions (M&A) of targeted firms, unless the prior firm performance has been either extremely high or low (Cho et al., 2016).

Although an award is a non-financial incentive that can motivate people, being celebrities will bring CEOs enormous financial benefits (Gallus and Frey, 2016; Shi et al., 2017). CEO’s social identity and influence will rise rapidly after winning business awards and getting the celebrity status (Hayward et al., 2004). As ambition for social recognition and celebrity status is a basic human instinct, CEOs are delighted to accept the celebrity status and also, the benefits that come with awards (Frey, 2007). First, celebrity CEOs can demand richer compensation packages after becoming celebrities (Graffin et al., 2008; Malmendier and Tate, 2009). Second, owing to their perceived power in the minds of stakeholders and the public, celebrity CEOs assume greater control over their firms (Cho et al., 2016). For instance, since directors usually do not have sufficient information about strategic decision and sufficient time for their board duties, they are inclined to trust celebrity CEOs who are considered to own good reputation and observe social norms, thus giving CEOs more power to implement the decisions they have chosen (Stevens, 2002; Yin et al., 2021). Third, their celebrity status helps them mobilize greater support for decisions that they make and provide more resources for their present and proposed actions (Wade et al., 2006). Finally, it provides advantages for recruitment of more valuable employees, developing relationships with suppliers, and obtaining extra financial resources (Hayward et al., 2004; Rindova et al., 2006). These benefits may encourage CEOs to cultivate such status enhancements and to take actions to preserve celebrity status (Cho et al., 2016).

From a psychological perspective, identity theory provides further interpretation of the impact of celebrity CEOs on firms’ behavior. It argues that an identity is built based on processes that have prompted humans to categorize, classify, or name themselves as part of certain social groups (Burke, 1991). The core of this identification process is categorizing an individual as someone who plays a specific social role that creates meanings and expectations for that role and associated behaviors (Thoits, 1986). These social roles have criteria that guide individual actions (Burke, 1991). If an individual does well in a specific role and gain a higher sense of self-esteem and self-efficacy, the results will be a higher level of self-consistency and self-regulation of behavior (Burke and Stets, 1999). In other words, people tend to preserve their identity when they perform well and feel comfortable while fulfilling a specific social role. However, according to social norm theory, if individual deviates from the expectations and norms of this specific role, punishment would be imposed both by people whose interests are affected and by third parties who are unaffected (Piskorski and Gorbatai, 2017; Blay et al., 2018). It may cause individuals to be separated from the social group they belong and lose the associated benefits (Yin et al., 2021).

Awards for CEOs become part of an identification process as celebrity CEOs, prompting the recipients to internalize the values and meanings attached to this celebrity identity and establish self-cognition consistent with the expectation from the public and the media (Owens et al., 2010; Lee et al., 2020). A celebrity CEO is usually an outstanding person who is better at business than most of his or her peers and tend to seek for collaboration only with similar counterparts, which further enhances self-consistency and self-regulation (Burke and Stets, 1999; Vatamanescu et al., 2020). Hence, celebrity CEOs tend to behave in a manner consistent with the celebrity identity that corresponds with the expected views of themselves and the stakeholders (Rindova et al., 2006; Zavyalova et al., 2017). Moreover, the public and the media are more likely to attribute excellent past performance to the celebrity CEO’s leadership rather than other factors that could have affected the overall standing of the firm in the market (Quigley and Hambrick, 2015; Lee et al., 2020). However, the downside of such attribution is that stakeholders and the media will routinely undeservedly blame celebrity CEO for failure and declining performance (Hayward et al., 2004; Graffin et al., 2008). Therefore, most celebrity CEOs carry a psychological burden in their role since the public and stakeholders invariably put pressure and expectation on them to continuously improve firm performance (Wade et al., 2006; Fralich and Papadopoulos, 2020). In particular, the greater the celebrity position of a CEO, the more closely the CEO is related to firm performance (Hayward et al., 2004; Graffin et al., 2008).

Meanwhile, celebrity status is not permanent, especially when the CEOs cannot keep or improve firm performance (Lovelace et al., 2018). If celebrity CEOs deviate from social norms and expectations that celebrity group should meet, they are in great danger of losing their existing identity and status (Yin et al., 2021). Such CEOs who have achieved a high level of performance have better understanding of the difficulties in achieving higher performance and the possible loss of the benefits offered by celebrity status (Lee et al., 2020). To preserve the eminent status, celebrity CEOs tend to take action that will help them maintain a consistently higher level of performance (Humphrey and Aime, 2014). There is a significant positive relationship between innovation input and firm performance (Wang et al., 2017; Lazaroiu et al., 2020). Innovation is the driving force for a firm’s survival and development. Firms can increase innovation input sustainably to obtain long-term competitive advantages, mitigate negative social influences, meet stakeholders’ expectations and achieve a substantial improvement in performance (Ballot et al., 2006; Lazaroiu et al., 2020). Therefore, under the pressure of performance expectations and identity psychological burden, celebrity CEOs are more likely to increase innovation investment to achieve better performance.

Furthermore, celebrity CEOs tend to become overconfident about their own abilities and strategic decisions that had brought about their celebrity status (Hayward et al., 2004). In some cases, celebrity status could generate overconfidence and enhance narcissistic behavior (Chatterjee and Hambrick, 2011), leading celebrity CEOs to trust that they are able to manage risky strategies (Cho et al., 2016). In particular, celebrity CEOs have great confidence in their abilities relevant to innovation strategy (Lovelace et al., 2018), and they may be more likely to overestimate the probability of success and profitability from investing in new products (Camerer and Lovallo, 1999). Previous research has suggested that there is a positive and significant correlation between overconfident CEOs and innovation input (Li and Zhang, 2022). Hence, we argue that celebrity CEOs are more likely to increase innovation investment, compared to non-celebrity CEOs. Thus, we propose the following hypothesis:

*H1:* Celebrity CEOs are positively related to innovation investment of firms.

### Moderating effect of analyst coverage

Securities analysts make evaluations and recommendations about firms and offer relevant information to investors (Hong et al., 2000). They influence not only the investors’ expectations and decisions, but also executives’ preferences and the strategic decisions of firms (Benner and Ranganathan, 2012). Prior studies have verified the impact of analyst coverage on organizational behavior and results. Analyst coverage promotes firms’ investment and financing decision and decreases information asymmetry and capital cost (Kelly and Ljungqvist, 2012; Derrien and Kecskes, 2013). When there is a reduction in analyst coverage, firms voluntarily disclose more information than mandated, and subsequently improves liquidity (Balakrishnan et al., 2014). Therefore, as important information intermediaries, analysts can play an external governance role and serve as an effective monitoring function (Bradley et al., 2022). Increasing analyst coverage leads to better financial reporting quality (Irani and Oesch, 2013), decreases in value-reducing acquisitions (Chen et al., 2015), and declines in earnings management (Yu, 2008).

From a principal-agent perspective, executives tend to have a negative attitude toward innovation strategy owing to the risky, uncertain, and long-term nature of innovation (Balkin et al., 2000). Conversely, investors prefer companies that excel in innovation activities (Gentry and Shen, 2013). Particularly, in China’s institutional environment, innovation is considered the primary driver of economy and prioritized by the government as a development strategy. Innovation-based firms can obtain substantial policy support, such as R&D subsidies and tax preferences (Genin et al., 2021). In addition to investors, analysts have higher enthusiasm and expectations for the innovation signals of firms (Frankel and Li, 2004). Sustainable innovation practice can be regarded as a signal of strong competitive advantage and positive future development, which is an effective assessment criterion for analysts (Kliestik et al., 2020; Lazaroiu et al., 2020). Greater analyst coverage brings about more supervision and lessens agency problems. A reduction in innovation investment, once discovered by analysts, may negatively influence the investors’ interest and the firm’s market value (Chauvin and Hirschey, 1993). To avoid bad evaluation from analysts, executives are likely to promote innovation input under greater analyst coverage (Gentry and Shen, 2013).

From the perspective of identity theory, greater analyst coverage may stimulate celebrity CEOs’ identity control mechanism which extends identity theory and argues that individuals perceive and internalize identity-related values and expectations when interacting with the external society (Burke, 1991). Hence, celebrity CEOs have sufficient motivation to change others’ views on themselves through identity control mechanisms if the external expectations change (Lee et al., 2020). Meanwhile, special attention from analysts creates higher expectation, leading to conflict between celebrity CEOs’ identity standards and actual self-view (Galvin et al., 2010). However, not meeting the analysts’ expectations is often considered as managerial failure and can create negative impact on the capital market (Qian et al., 2019). Moreover, greater analyst coverage strengthens the association between firm performance and CEO turnover (Farrell and Whidbee, 2003). Facing greater analyst coverage of their firms, celebrity CEOs recognize that there are higher standards for maintaining their identity and status with the public as well as their own self-identity (Humphrey and Aime, 2014). Consequently, celebrity CEOs will take actions to reduce the dissonance between their individual performance and external expectations, in case of the loss of celebrity identity (Stets and Burke, 2000). The CEOs’ subsequent decisions and behaviors will be reflected in firms’ strategies (Cho et al., 2016). They will become more daring in terms of investments in innovative practices to satisfy the needs of analysts and investors and further improve firm performance. In light of these arguments, we put forward the following hypothesis:

*H2:* Analyst coverage positively moderates the relationship between celebrity CEOs and firms’ innovation investment.

### Moderating effect of the nature of firm ownership

Firms’ behaviors and strategies are closely related to the nature of enterprise ownership (Gelfand et al., 2007). In China, SOEs are an important part of national economy and bear a large number of policy burdens to support governmental policies (Wei, 2021). SOEs are mainly distributed in pillar industries and controlled by the state through the State-owned Assets Supervision and Administration Commission of the State Council (SASAC), which has the power to appoint and remove SOEs’ directors and executives (Bruton et al., 2015). Thus, CEOs in SOEs have to consider the general policy of government and SASAC’s orientation when he/she develops and implements his or her firm’s strategies (Bai et al., 2006). Hence, it inevitably leads to a decline in the influence of celebrity CEOs’ individual motivation on firm decision-making (Li and Zhang, 2022).

Additionally, as the business objectives and governance mechanisms of different ownership enterprises in China are various, CEOs have different degrees of innovation willingness (Li, 2013). Unlike non-SOEs, the operating goal of SOE is not entirely profit maximization (Ghosh and Whalley, 2008). The government set various sociopolitical goals to SOEs, such as job creation, infrastructure development and maintenance, improving public services, contributing to social welfare, and maintaining social stability (Bai and Xu, 2005). SOEs’ executives has a dual identity: economic and political, as they are not only firms’ top managers, but also officials in the government. There are personnel circulation channels between SOEs and government departments, which allow managers and officials to realize identity exchange. Therefore, the assessment and incentive mechanisms of executives in SOEs are not completely related to firm performance (Bruton et al., 2015). Different form non-SOEs, the development of their career path and political future depends on the realization of social goals and political missions (Shao et al., 2020). These CEOs are more driven to maintain their political identity by completing established social or political tasks, since the political identity is more conducive to personal interests and future development in the Chinese institutional environment. Moreover, the political burden in SOEs lead executives to waste extra resources to enhance their political performance at the expense of innovation activities (Song et al., 2015; Bertrand et al., 2018). Consequently, without performance pressure, Celebrity CEOs in SOEs do not need to maintain their celebrity status through excellent firm performance, leading to weak motivation to increase innovation investment. Further, the dual identities of SOEs’ managers prompt them to become more involved in low-risk projects with short-term payoffs, at the expense of a decrease in long-term and higher risk innovation activities and R&D investment (Wang et al., 2018). Owing to the lack of ownership, it is difficult for SOEs to supervise executives effectively, which further exacerbates their tendency of risk aversion. Substantial evidence from previous research shows that CEOs with more political connections reduce R&D intensity and innovation efficiency (Lin et al., 2014; Zhang et al., 2015).

Some studies have demonstrated the inefficiency of government participation in terms of corporate innovation, with R&D efficiency being higher in non-SOEs than in SOEs (Zhou et al., 2017; Zhang et al., 2020; Genin et al., 2021). Zhang et al. (2020) used the sample of listed companies in Chinese manufacturing industry from 2011 to 2015 to demonstrate that most SOEs’ size are higher than private-owned enterprises (non-SOEs), but the growth, profitability and R&D intensity of non-SOEs are better. They found that the average R&D intensity, which is measured as the ratio of R&D expenses and total asset, is 0.018 in SOEs, lower than 0.021 in non-SOEs. For comparison, we divide our sample firms into SOE and non-SOE sub-samples, and measure R&D intensity with the same measurement method. The R&D intensity is 0.018 in SOEs, lower than 0.024 in non-SOEs, meaning that the difference between SOEs and non-SOEs in innovation investment is huge. Private enterprises are more independently operated than SOEs, and their CEOs have more power in firm’s daily operations and decision-making (Li and Zhang, 2022). Hence, in the private sector, the impact of celebrity CEOs’ personal motivation and decision preference on firm strategies faces fewer constraints and restrictions.

Although the impact of central planning has greatly diminished with the deepening of reform and opening-up, government as the most authoritative institution, still actively leads economic development and constantly adjust firms’ environment through regulatory policies and allocation of scarce resources (Liang et al., 2015; Zhou et al., 2017). In this institutional context, the nature of state-owned property of firms can exert crucial influence on innovation strategy, since the state provides more innovation resources to SOEs than to other firms (Bruton et al., 2015; Ramamurti and Hillemann, 2018; Hu et al., 2019). SOEs are able to receive resources, such as financial and human capital, research funding, bank lending, technological equipment procurement, national research and knowledge platform, access to specific industry and other government policy benefits, which are all necessary for innovation activities (Li et al., 2018; Liu et al., 2018). State ownership and its advantages not only reduce the transformation efficiency from plentiful resource into innovation investment (Zhou et al., 2017; Genin et al., 2021), but also weaken celebrity CEOs’ important role of providing additional resources to their firms. Additionally, state affiliation of firms is more likely to restrict the integration and utilization of CEOs’ social resources, thus negating the benefits of CEOs’ celebrity status on innovation investment (Li et al., 2018). Unlike SOEs, non-SOEs often lack of access to innovation resources and face relatively higher innovation barriers such as asymmetrical information and financial constraints (Howell, 2016). To achieve innovation goals, non-SOEs typically seek more social resources than political ones, which highlights celebrity CEOs’ resource advantage (Kroll and Kou, 2019; Lazzarini et al., 2021). CEOs’ celebrity status as a kind of intangible resource can provide more resources for innovation strategy (Lee et al., 2020; Kim and Lee, 2022), and non-SOEs have been shown to be more efficient in terms of resource utilization (Howell, 2020). Therefore, non-SOEs tend to efficiently transform these valuable scarce resources brought by celebrity CEOs into innovative investment. In light of these arguments, we expect that the association between celebrity CEOs and innovation investment is more significant in Chinese non-SOEs. Therefore, we develop the following hypothesis:

*H3:* Compared to SOEs, celebrity CEOs of non-SOEs have a more significant impact on innovation investment.

## Materials and methods

### Sample selection and data sources

This study uses a panel dataset of firms listed on the Shanghai and Shenzhen stock exchanges in China from 2015 to 2020. The vast majority of Chinese listed companies are concentrated in Shanghai and Shenzhen stock exchanges. And such listed firms are more mature and stable with better transparent data disclosure. They are also larger and more probable for recruiting or cultivating celebrity CEOs. So, the selection of firms listed on the Shanghai and Shenzhen stock exchanges is universal and representative. In 2015, the Chinese government introduced specific guidance documents to promote the innovative development strategy that was the most important policy to encourage firms’ innovation activities in recent decades. After that, the innovation in Chinese firms are facing a new era of major development and major changes. In order to cope with the changes of external institutional environment, innovation input are crucial and significantly increased in Chinese firms during this period. Therefore, we chose 2015 as the starting point of the sample interval. Meanwhile, there is a one-year lag between the independent and dependent variables. Since 2021 is the last year for available data of Chinese listed firms, we chose 2020 as the end point.

Data of celebrity CEOs were hand-collected from resumes, personal profiles, corporate annual reports, firm websites, social media, news reports, and search engines on the Internet. Other CEO information and firm-level data were collected from China Stock Market and Accounting Research Database (CSMAR) as well as databases of the Shanghai and Shenzhen Stock Exchanges. First, we excluded financial and insurance companies because of the particularity and complexity of financial indices and operational objectives. Second, firms whose data on key variables were incomplete were omitted from the study. Then, we eliminated samples where the CEOs’ tenure was less than 12 months, as such a CEO would have had little time to exert influence over the company’s strategies and operations. Afterwards, we omitted listed companies under special treatment to avoid the impact of extreme values on the analysis results. Our final sample comprised 10,677 firm-years. Finally, we winsorized all continuous variables at the top and bottom 1% of the sample to eliminate the effect of outliers.

### Measures

#### Dependent variable

*Innovation investment.* Our main hypotheses addressed the impact of celebrity CEOs on innovation investment, which were measured as the expenditures spent by a firm on innovative practices. Considering the heterogeneity in terms of the company size, R&D intensity was widely used to measure innovation investment, estimated as R&D expenditures divided by operation revenue (Heyden et al., 2017). The existing literature has shown that firms’ innovation input lags behind strategic decisions, and the impact of CEO’s personal characteristics on decision-making also has a significant lag (Wal et al., 2019). Hence, a lag of one period is adopted to treat the dependent variable.

#### Independent variable

*CEO celebrity.* Previous studies have defined celebrity status using the results of annual CEO award competitions from various prestigious business journals, including Financial World, Business Week, Chief Executive, and Morningstar (Lee et al., 2020). Winning a business award from the media provides a reliable assessment of the CEO by a group of experts in society and business, which can accurately capture the celebrity status (Wade et al., 2006; Malmendier and Tate, 2009; Cho et al., 2016). Based on this measurement, we used relevant top awards issued by authoritative business media as a proxy, including China Central Television (CCTV), Finance Channel, Forbes China, China Business Channel, Fortune China, and China Times. Further, we extended the current measurement method by considering China’s unique institutional environment. In the Chinese context, awards issued by government departments are far more persuasive and influential than those issued by the media, such as “Outstanding Entrepreneurs,” “Model Worker,” and “Outstanding Youth..” Therefore, we incorporated national and provincial top honorary awards into the measurement to expand its applicability in the Chinese context. Then, being a celebrity CEO was measured as a dummy variable, which was coded as 1 if the CEO had an award before focal year t, and 0 otherwise.

#### Moderating variables

*Analyst coverage. A*nalyst coverage was measured as the number of securities analysts who issued earnings forecasts for sample firms during the study’s period (Gentry and Shen, 2013). To overcome the data skewness, the natural logarithm was taken after adding 1 to the obtained data. A higher value of this measure indicated a higher level of analyst coverage.

*Nature of firm Ownership.* The nature of firm ownership was estimated as a dummy variable. If the actual controller of a sample firm was the state or an institution representing the state, we coded it as 1; otherwise, 0 (Wang et al., 2022).

#### Control variables

Drawing on previous studies on CEO individual characteristics and innovation investment, we employed control variables from firm financial level, corporate governance level, and CEO individual level (Galasso and Simcoe, 2011; Wang et al., 2017; Rodrigues et al., 2020; Zhang et al., 2021). The firm-financial-level control variables included the return on total assets (ROA), total assets turnover (ATO), cash flow ratio (CFR), liabilities-to-assets ratio (Lev), firm size (FS), and firm age (FA). The corporate-governance-level control variables included board size (BS), board independence (BI), large shareholders’ control (LSC), and the pay gap of top management (Gap). The CEO-individual-level control variables included CEO gender (Gender), CEO age (Age), and CEO’s political connection (PC). We also controlled for year and industry fixed effects. The above variables and explanations are shown in Table 1.

**Table 1:** Definition and measurement of variables.

Variable type	Variable name	Symbol	Measurement method
Dependent variable	R&D intensity	RD	R&D investment/operating income
Independent variable	Celebrity CEO	CCEO	CEO won an award = 1, CEO not won an award = 0
Moderating variables	Analyst Coverage	AC	Ln (number of analysts cover the firm +1)
	Nature of firm ownership	SOE	State-owned enterprises = 1, non-state-owned enterprises = 0
Control variables	Return on total assets	ROA	Net profit/total assets
	Total assets turnover	ATO	Operating income/total assets
	Cash flow ratio	CFR	Operating cash flow/total assets
	Asset-liability ratio	Lev	Total liabilities/total assets
	Firm size	FS	Ln (total assets)
	Firm age	FA	Ln (Actual firm age of the year)
	Board size	BS	Ln (Number of formal members of the board of directors)
	Board independence	BI	Proportion of independent directors
	Large shareholder control	LSC	Number of shares held by the largest shareholder/total shares
	Pay gap of top management	Gap	Sum of top three executives’ compensation/all executives’ compensation
	CEO gender	Gender	Male = 1, Female = 0
	CEO age	Age	Ln (Actual CEO age in the year)
	CEO’s political connection	PC	CEO hold or previously held a position in the government =1, otherwise = 0

### Model construction

To test the impact of celebrity CEO on innovation investment as well as the moderating effects of analyst coverage and nature of firm ownership, we constructed and employed the following models:


(1) $$RD_{t+1}=\alpha_{0}+\alpha_{1}CCEO_{t}+\sum \mathrm{\alpha Control}s_{t}+\varepsilon$$

(2) $$\begin{array}{l} RD_{t+1}=\beta_{0}+\beta_{1}CCEO_{t}+\beta_{2}AC_{t}+\beta_{3}CCEO_{t}\\ \;\times AC_{t}+\sum \mathrm{\beta Control}s_{t}+\varepsilon \end{array}$$

Among them,α and β represent the coefficients of each variable. Controls indicate all the control variables. CCEO∗AC denotes the interaction term between celebrity CEOs and analyst coverage. Model (1) was employed to test the effect of celebrity CEO on innovation investment, and model (2) was used to estimate the moderating effect of analyst coverage on the relationship between celebrity CEO and innovation investment. To assess the moderating effect of the nature of firm ownership, we used model (1) to test SOEs and non-SOEs separately.

## Results

### Descriptive statistics

Table 2 reports descriptive statistical results of the main variables. As shown in Table 2, the mean value of R&D intensity (RD) is 0.051, the minimum value is 0, and the maximum value is 5.452, indicating substantial differences in innovation investment for different firms. The mean value for celebrity CEOs (CCEO) is 0.066, indicating that only few CEOs obtain awards issued by top business media and the government. The mean value of analyst coverage (AC) is 1.493, the minimum value is 0, and the maximum value is 4.331, indicating significant differences in analyst coverage among the samples. The ratio of SOE in our sample is 0.328, indicating that about a third of the sample firms are SOEs.

**Table 2:** Descriptive statistical results of variables.

Variable	Obs.	Mean	S.D.	Min	Max
RD	13,546	0.051	0.089	0	5.452
CCEO	13,546	0.066	0.248	0	1
AC	13,546	1.493	1.135	0	4.331
SOE	13,546	0.328	0.469	0	1
ROA	13,546	0.047	0.060	−0.389	0.244
ATO	13,546	0.642	0.398	0.044	2.777
CFR	13,546	0.047	0.066	−0.196	0.258
Lev	13,546	0.393	0.198	0.046	0.990
FS	13,546	22.070	1.235	18.330	28.540
FA	13,546	2.833	0.326	1.792	3.555
BS	13,546	2.118	0.194	1.609	2.708
BI	13,546	0.376	0.053	0.308	0.600
LSC	13,546	0.340	0.144	0.084	0.755
Gap	13,546	0.456	0.166	0	1
Gender	13,546	0.938	0.241	0	1
Age	13,546	3.901	0.137	3.258	4.382
PC	13,546	0.188	0.391	0	1

### Correlation analysis

Table 3 shows the Pearson test on the correlation of main variables. The absolute value of correlation coefficients between all variables is below 0.6, indicating the suitability of using these variables in our models simultaneously. We also estimated the mean variance inflation factor (VIF) for regression analysis. The maximum VIF value is 1.86, far below 10, indicating very limited multicollinearity.

**Table 3:** Correlation analysis results.

	1	2	3	4	5	6	7	8	9	10	11	12	13	14	15	16	17
1.RD	1																
2.CCEO	0.023***	1															
3.AC	0.014*	0.072***	1														
4.SOE	0.120***	−0.003	0.030***	1													
5.ROA	−0.022**	0.023***	0.236***	0.188***	1												
6.ATO	−0.181***	0.015*	0.084***	−0.069***	0.184***	1											
7.CFR	−0.041***	0.023***	0.142***	0.040***	0.420***	0.142***	1										
8.Lev	−0.186***	0.039***	0.017*	−0.329***	−0.390***	0.169***	−0.173***	1									
9.FS	−0.148***	0.091***	0.243***	−0.395***	−0.070***	0.091***	0.045***	0.543***	1								
10.FA	−0.073***	0.007	−0.045***	−0.209***	−0.075***	0.029***	0.025***	0.146***	0.165***	1							
11.BS	−0.063***	0.000	0.053***	−0.268***	−0.036***	0.032***	0.014	0.168***	0.275***	0.077***	1						
12.BI	0.026***	0.032***	0.009	0.055***	0.003	−0.029***	0.005	−0.022**	−0.021**	−0.041***	−0.566***	1					
13.LSC	−0.098***	−0.024***	0.020**	−0.217***	0.092***	0.097***	0.093***	0.062***	0.174***	−0.070***	0.004	0.056***	1				
14.Gap	0.044***	0.015*	−0.060***	0.007	0.032***	0.001	0.097***	−0.103***	−0.139***	0.155***	−0.223***	0.100***	−0.008	1			
15.Gender	0.001	−0.004	0.011	−0.058***	−0.014	0.029***	−0.026***	0.044***	0.056***	−0.017**	0.081***	−0.062***	−0.021**	−0.067***	1		
16.Age	0.005	0.063***	0.022**	−0.105***	0.002	−0.009	0.043***	0.018**	0.098***	0.120***	0.050***	0.012	0.041***	0.054***	0.030***	1	
17.PC	−0.024***	0.103***	0.027***	0.102***	0.018**	−0.020**	0.016*	−0.018**	−0.013	−0.006	−0.016*	0.028***	−0.017**	0.021**	−0.043***	0.058***	1

*p < 0.1, **p < 0.05; ***p < 0.01.

### Empirical results

Table 4 presents the regression results of fixed-effect analyses controlling for year and industry. As shown in the first column of Table 4, the correlation coefficient between celebrity CEOs and innovation investment is 0.0075 and significantly positive at the 1% level. That is, R&D intensity is stronger in firms controlled by celebrity CEOs, indicating that celebrity CEOs increase firms’ innovation investment. Hypothesis 1 is thus supported.

**Table 4:** Regression analysis results.

Variables	RD_t + 1_			
	(1)	(2)	(3)	(4)
			SOEs	Non-SOEs
CCEO	0.0075***	0.0006	0.0069**	0.0077***
	(4.46)	(0.22)	(2.33)	(3.92)
AC		0.0042***		
		(5.17)		
CCEO*AC		0.0035***		
		(2.63)		
ROA	−0.0981	−0.1163*	−0.0632	−0.1149
	(−1.52)	(−1.73)	(−0.89)	(−1.29)
ATO	−0.0263***	−0.0267***	−0.0295***	−0.0247***
	(−17.37)	(−17.62)	(−8.37)	(−18.60)
CFR	−0.0189*	−0.0216*	−0.0226	−0.0166
	(−1.65)	(−1.86)	(−1.04)	(−1.25)
Lev	−0.0640***	−0.0630***	−0.0626***	−0.0646***
	(−7.51)	(−7.49)	(−3.68)	(−6.85)
FS	−0.0014*	−0.0027***	−0.0011	−0.0015
	(−1.71)	(−3.13)	(−0.79)	(−1.56)
FA	−0.0164***	−0.0154***	−0.0131	−0.0180***
	(−3.88)	(−3.74)	(−1.38)	(−4.22)
BS	−0.0011	−0.0008	−0.0066	0.0013
	(−0.38)	(−0.28)	(−1.28)	(0.37)
BI	0.0147	0.0140	0.0318	0.0053
	(1.16)	(1.10)	(1.52)	(0.31)
LSC	−0.0172*	−0.0151	−0.0274***	−0.0120
	(−1.76)	(−1.51)	(−2.88)	(−0.87)
Gap	−0.0119**	−0.0118**	−0.0061	−0.0145**
	(−2.35)	(−2.33)	(−0.76)	(−2.26)
Gender	0.0017	0.0017	0.0017	0.0014
	(0.92)	(0.93)	(0.53)	(0.66)
Age	0.0140**	0.0137**	0.0212	0.0100
	(2.18)	(2.14)	(1.42)	(1.61)
PC	−0.0051***	−0.0053***	−0.0020	−0.0066***
	(−2.95)	(−3.06)	(−0.45)	(−4.79)
Constant	0.0850***	0.1050***	0.0486	0.1052***
	(3.41)	(4.16)	(0.84)	(4.11)
Industry	Yes	Yes	Yes	Yes
Year	Yes	Yes	Yes	Yes
R^2^	0.133	0.136	0.115	0.146
N	13,546	13,546	4,442	9,104

Robust standard errors in parentheses. *p < 0.1, **p < 0.05, ***p < 0.01.

The second column reports the moderating effect of analyst coverage on the relationship between celebrity CEO and innovation investment. The interaction term coefficient is 0.0035 and significantly positive at the 1% level. This indicates that the promoting effect of celebrity CEOs on R&D intensity could be strengthened with the increase in a firm’s analyst coverage. That is, analyst coverage plays a positive moderating role between celebrity CEO and innovation investment. Hypothesis 2 is thus supported.

Furthermore, we examined the differences in the impact of celebrity CEOs on innovation investment among firms with different ownership natures. The third and fourth columns report the regression results for celebrity CEO and R&D intensity in SOEs and non-SOEs separately. For the SOEs sample, the correlation coefficient between celebrity CEO and R&D intensity is 0.0069 and positively significant at the 5% level. Meanwhile, for the non-SOE sample, the correlation coefficient is 0.0077 and significantly positive at the 1% level. As both correlation coefficient and significance level of the non-SOEs groups are higher than those of the SOEs’ group, it indicates that the promoting effect of celebrity CEOs on innovation investment is more significant in non-SOEs, thus supporting Hypothesis 3.

### Robustness tests

#### Propensity score matching method

Since firms with strong willingness to innovate strategy may be more likely to hire a celebrity CEO than other firms, the endogenous choice of celebrity CEOs are more likely to have affected the analysis results. Following previous studies, we employed the propensity score matching (PSM) method to solve this potential problem. For each firm with a celebrity CEO, we identified a matched control firm without a celebrity CEO and calculated the average difference in R&D intensity for all matched pairs. To find the matched firms, we employed a 1:4 nearest neighbor matching technique. Our matching covariates included all control variables in the baseline model. We used the treated group and matched samples data to rerun the whole model. Table 5 reports the results of the PSM robustness test. There is a positive association between Celebrity CEOs and firms’ innovation investment. And the promotion effect of celebrity CEOs on innovation investment is also positively moderated by analyst coverage. The correlation coefficient of celebrity CEOs in the SOE group is less than in the non-SOEs group, indicating that the promoting effect of celebrity CEOs on innovation investment is more significant in non-SOEs. The results ensure the robustness of the baseline regression of this study.

**Table 5:** Robustness test: Propensity score matching analysis.

Variables	RD_t + 1_			
	(1)	(2)	(3)	(4)
			SOEs	Non-SOEs
CCEO	0.0076***	0.0071**	0.0077**	0.0121***
	(4.28)	(2.13)	(2.42)	(5.17)
AC		0.0041***		
		(4.56)		
CCEO*AC		0.0031*		
		(1.89)		
ROA	0.0005	−0.0109	−0.0349	0.0339
	(0.02)	(−0.35)	(−0.66)	(0.91)
ATO	−0.0228***	−0.0283***	−0.0214***	−0.0283***
	(−12.92)	(−16.91)	(−6.59)	(−14.47)
CFR	−0.0031	−0.0285*	−0.0080	−0.0224
	(−0.22)	(−1.88)	(−0.36)	(−1.16)
Lev	−0.0440***	−0.0477***	−0.0318***	−0.0549***
	(−5.91)	(−6.21)	(−2.99)	(−5.40)
FS	−0.0011	−0.0049***	−0.0020	−0.0029***
	(−1.43)	(−5.97)	(−1.45)	(−3.05)
FA	−0.0108***	−0.0129***	−0.0064	−0.0148***
	(−4.29)	(−4.79)	(−1.25)	(−4.59)
BS	−0.0060	−0.0047	−0.0214**	0.0017
	(−1.36)	(−0.96)	(−2.56)	(0.29)
BI	0.0324**	0.0435***	0.0354	0.0417**
	(2.27)	(2.71)	(1.35)	(2.10)
LSC	−0.0263***	−0.0412***	−0.0300***	−0.0445***
	(−4.82)	(−6.87)	(−2.94)	(−6.19)
Gap	−0.0029	−0.0098	0.0088	−0.0165*
	(−0.40)	(−1.28)	(0.61)	(−1.85)
Gender	0.0015	0.0030	−0.0017	0.0052
	(0.58)	(0.96)	(−0.37)	(1.38)
Age	0.0097*	−0.0001	0.0237**	−0.0078
	(1.67)	(−0.02)	(2.29)	(−1.02)
PC	−0.0046***	−0.0094***	−0.0049	−0.0090***
	(−2.84)	(−5.45)	(−1.54)	(−4.38)
Constant	0.0684**	0.2279***	0.0403	0.2155***
	(2.26)	(7.21)	(0.85)	(5.34)
Industry	Yes	Yes	Yes	Yes
Year	Yes	Yes	Yes	Yes
R^2^	0.317	0.380	0.307	0.183
N	3,747	3,747	1,208	2,539

Robust standard errors in parentheses. *p < 0.1, **p < 0.05, ***p < 0.01.

#### Two-stage least squares method

We employed the Two-Stage Least Squares (2SLS) method to solve the endogeneity problem. We used advertising expenditure as an instrument variable in our model, which was measured as the ratio of annual advertising expenses to revenue. More advertising expenditure will not only reduce the use of negative words on firms and their leaders in the media, but also encourage the media to help CEOs attract the attention and favor of the society and improve their possibility of winning awards and being celebrities (Gurun and Butler, 2012; Beattie et al., 2021). Also, advertising expenditure is exogenous to the innovation input. While running the first-stage regression analysis, the instrument variable shows significant correlation with celebrity CEOs but not with the R&D intensity, which ensures that it could be an effective instrument. Table 6 reports the results of the second-stage 2SLS model. There is a positive association between celebrity CEOs and firms’ innovation investment. The analyst coverage strengthens the promotion effect of celebrity CEOs on innovation investment. For the results of grouping regression, the correlation coefficients of celebrity CEOs are both significant in SOE group and non-SOE group. But the correlation coefficient is larger for non-SOEs than for SOEs. The results are consistent with the baseline regression results, and they pass the underidentification and weak identification test, indicating that endogeneity is not a relevant concern in this study.

**Table 6:** Robustness test: Two-stage least squares analysis.

Variables	RD_t + 1_			
	(1)	(2)	(3)	(4)
			SOEs	Non-SOEs
CCEO	0.0136***	0.0042	0.0123***	0.0142***
	(5.20)	(0.85)	(2.82)	(4.37)
AC		0.0042***		
		(5.67)		
CCEO*AC		0.0049**		
		(2.14)		
ROA	−0.0730	−0.0921	−0.0471	−0.0855
	(−1.16)	(−1.40)	(−0.67)	(−0.99)
ATO	−0.0295***	−0.0299***	−0.0305***	−0.0290***
	(−27.85)	(−28.29)	(−15.36)	(−23.56)
CFR	−0.0315***	−0.0339***	−0.0349*	−0.0297**
	(−3.11)	(−3.32)	(−1.82)	(−2.55)
Lev	−0.0694***	−0.0682***	−0.0672***	−0.0704***
	(−7.53)	(−7.50)	(−3.79)	(−6.75)
FS	−0.0024***	−0.0036***	−0.0023	−0.0024***
	(−2.90)	(−4.27)	(−1.50)	(−2.59)
FA	−0.0150***	−0.0138***	−0.0126	−0.0161***
	(−4.61)	(−4.34)	(−1.61)	(−5.36)
BS	−0.0045	−0.0043	−0.0111**	−0.0015
	(−1.48)	(−1.40)	(−2.17)	(−0.41)
BI	0.0187	0.0174	0.0379*	0.0084
	(1.39)	(1.29)	(1.76)	(0.46)
LSC	−0.0417***	−0.0395***	−0.0516***	−0.0367***
	(−4.68)	(−4.32)	(−5.18)	(−2.98)
Gap	0.0175***	0.0183***	0.0139	0.0193***
	(4.20)	(4.36)	(1.63)	(4.10)
Gender	0.0042**	0.0043**	0.0030	0.0047**
	(2.19)	(2.21)	(0.86)	(2.02)
Age	0.0118*	0.0118*	0.0174	0.0089
	(1.73)	(1.73)	(1.18)	(1.28)
PC	−0.0081***	−0.0084***	−0.0053	−0.0093***
	(−4.84)	(−5.00)	(−1.25)	(−6.78)
Constant	0.1560***	0.1743***	0.1379**	0.1660***
	(5.80)	(6.49)	(2.18)	(6.65)
Industry	Yes	Yes	Yes	Yes
Year	Yes	Yes	Yes	Yes
R^2^	0.301	0.303	0.277	0.315
N	13,546	13,546	4,442	9,104

Robust standard errors in parentheses. *p < 0.1, **p < 0.05, ***p < 0.01.

#### Replacing the measure of innovation investment

In the main test, the innovation investment was measured as R&D expenditures divided by operation revenue. Following prior research, we employed another measurement of innovation investment: R&D expenditures divided by a firm’s total assets (Lin et al., 2011). The variable was also treated with a 1-year lag. We then reconducted all base models. Table 7 reports the regression results of replacing the measure of innovation investment. The correlation coefficient between celebrity CEOs and innovation investment remains significantly positive. The interaction term of celebrity CEOs and analyst coverage remains significantly positive as well. The correlation coefficients between celebrity CEO and innovation for both SOEs and non-SOEs are significantly positive. However, the correlation coefficient is larger for non-SOEs than for SOEs. These results are consistent with our primary analysis.

**Table 7:** Robustness test: Replacing the measure of innovation investment.

Variables	RD_t + 1_			
	(1)	(2)	(3)	(4)
			SOEs	Non-SOEs
CCEO	0.0049***	0.0019	0.0044***	0.0052***
	(6.87)	(1.59)	(3.74)	(5.73)
AC		0.0016***		
		(8.78)		
CCEO*AC		0.0015**		
		(2.47)		
ROA	0.0148*	0.0079	0.0224**	0.0112
	(1.77)	(0.91)	(2.18)	(0.99)
ATO	0.0087***	0.0085***	0.0072***	0.0094***
	(12.63)	(12.45)	(4.99)	(13.41)
CFR	0.0161***	0.0151***	0.0229***	0.0129***
	(4.43)	(4.17)	(4.22)	(2.71)
Lev	−0.0100***	−0.0096***	−0.0093***	−0.0104***
	(−5.50)	(−5.34)	(−2.79)	(−4.95)
FS	−0.0018***	−0.0023***	−0.0021***	−0.0016***
	(−8.19)	(−10.67)	(−6.52)	(−5.89)
FA	−0.0030***	−0.0027***	−0.0014	−0.0038***
	(−4.55)	(−4.04)	(−1.10)	(−4.96)
BS	0.0019	0.0020	−0.0009	0.0030**
	(1.53)	(1.63)	(−0.37)	(2.09)
BI	0.0128***	0.0125***	0.0129*	0.0119**
	(3.22)	(3.16)	(1.80)	(2.51)
LSC	−0.0072***	−0.0064***	−0.0083***	−0.0066***
	(−4.39)	(−3.87)	(−2.68)	(−3.44)
Gap	−0.0056***	−0.0055***	−0.0075***	−0.0046**
	(−3.63)	(−3.60)	(−2.96)	(−2.36)
Gender	0.0017**	0.0017***	0.0026**	0.0012
	(2.56)	(2.58)	(2.29)	(1.56)
Age	0.0029**	0.0028**	0.0019	0.0032**
	(2.32)	(2.24)	(0.87)	(2.15)
PC	−0.0013**	−0.0014**	0.0005	−0.0022***
	(−2.25)	(−2.40)	(0.33)	(−4.72)
Constant	0.0342***	0.0418***	0.0460***	0.0295***
	(5.61)	(6.86)	(4.33)	(3.95)
Industry	Yes	Yes	Yes	Yes
Year	Yes	Yes	Yes	Yes
R^2^	0.221	0.227	0.186	0.250
N	13,473	13,473	4,424	9,049

Robust standard errors in parentheses. *p < 0.1, **p < 0.05, ***p < 0.01.

#### Additional lagged effects

The dependent variable was lagged for one period in our baseline models. However, it may take more time for CEOs to influence firms’ innovation strategy and increase innovation investment. Therefore, we added a two-year lag to the dependent variable before reconducting the regression. Table 8 reports the regression results of the additional lagged effects. There is a significant positive correlation between celebrity CEOs and innovation investment. Additionally, the interaction term of celebrity CEO and analyst coverage remains significantly positive. The correlation coefficient between celebrity CEOs and innovation for non-SOEs is greater than for SOEs. Therefore, all robustness tests support the results of our main analysis.

**Table 8:** Robustness test: Additional lagged effects.

Variables	RD_t + 2_			
	(1)	(2)	(3)	(4)
			SOEs	Non-SOEs
CCEO	0.0108***	0.0054*	0.0090***	0.0119***
	(5.88)	(1.70)	(2.97)	(4.99)
AC		0.0034***		
		(5.19)		
CCEO*AC		0.0027*		
		(1.79)		
ROA	0.0282	0.0094	0.0278	0.0279
	(0.74)	(0.23)	(0.70)	(0.52)
ATO	−0.0250***	−0.0252***	−0.0238***	−0.0256***
	(−13.27)	(−13.42)	(−10.04)	(−10.08)
CFR	0.0031	0.0018	−0.0008	0.0057
	(0.15)	(0.08)	(−0.04)	(0.19)
Lev	−0.0411***	−0.0406***	−0.0374***	−0.0432***
	(−6.19)	(−6.16)	(−3.93)	(−4.89)
FS	−0.0026***	−0.0036***	−0.0030***	−0.0025***
	(−3.62)	(−4.43)	(−2.94)	(−2.60)
FA	−0.0036	−0.0028	−0.0050	−0.0029
	(−1.43)	(−1.09)	(−1.23)	(−0.88)
BS	0.0006	0.0008	0.0008	0.0011
	(0.20)	(0.27)	(0.17)	(0.29)
BI	0.0250**	0.0245**	0.0335*	0.0220
	(2.20)	(2.17)	(1.78)	(1.56)
LSC	−0.0231***	−0.0215***	−0.0331***	−0.0186***
	(−5.67)	(−5.28)	(−5.18)	(−3.65)
Gap	−0.0001	0.0000	0.0013	−0.0015
	(−0.03)	(0.00)	(0.17)	(−0.25)
Gender	0.0046***	0.0046***	0.0049**	0.0043*
	(2.62)	(2.64)	(2.00)	(1.84)
Age	0.0020	0.0020	0.0028	0.0011
	(0.60)	(0.60)	(0.42)	(0.29)
PC	−0.0029	−0.0030	−0.0021	−0.0032
	(−1.53)	(−1.61)	(−0.65)	(−1.34)
Constant	0.1030***	0.1170***	0.1057***	0.1036***
	(5.00)	(5.48)	(2.80)	(4.25)
Industry	Yes	Yes	Yes	Yes
Year	Yes	Yes	Yes	Yes
R^2^	0.179	0.182	0.225	0.165
N	10,550	10,550	3,452	7,098

Robust standard errors in parentheses. *p < 0.1, **p < 0.05, ***p < 0.01.

## Discussion and conclusion

Some previous studies have argued that the celebrity status of CEOs have a positive impact on firms by attracting social attention and enhancing the prestige of organizations, leading to greater investor confidence, the acquisition of extra resources, and an increase in stock prices (Fralich and Papadopoulos, 2020; Lee et al., 2020; Kim and Lee, 2022). Celebrity CEOs also result in distinctive firm decisions and behaviors, including managerial risk-taking, acquisition premiums, and corporate social responsibility, which influence firm performance (Cho et al., 2016; Shi et al., 2017; Wei et al., 2018). However, few studies have explored firms’ innovation investment from the perspective of CEOs’ celebrity identity and status. Moreover, most studies used samples in developed countries and regions. Therefore, extending the line of current research, we investigated the effect of celebrity status on firms’ innovation investment in the context of China’s economic and institutional environment. Further, we explored the moderating effect of analyst coverage and ownership nature on the relationships between celebrity CEO and innovation investment.

Consistent with our theoretical arguments, we found that celebrity CEOs tend to support more innovation investment as a means of maintaining and promoting their identity and status as celebrities compared to CEOs without the celebrity label. Since celebrity CEOs have to act consistently with their identity standard as an outstanding entrepreneur after internalizing roles and expectations attached to the celebrity (Zavyalova et al., 2017; Lee et al., 2020). Otherwise, they are more likely to be punished by social norms, and may lose their celebrity identities and the huge benefits coming with it Innovation activities can not only capture public attention and improve the corporate reputation, but have also proven to be an effective tool for improving firm performance and obtaining future advantages for the company (Camison and Villar-Lopez, 2014; Nguyen et al., 2019). Therefore, celebrity CEOs have strong motivation to invest more in R&D for preserving their social status. Simultaneously, greater analyst coverage create more expectations and monitoring, lead celebrity CEOs to perceive more pressures and invest more in innovative activities because of self-protection motives. We also observed that the influence of CEO celebrity status on innovation investment tend to dissipate in SOEs. That is because the firms’ state-owned nature and executives’ dual identity as both executives and officials prompt CEOs to focus on political goals rather than performance (Bruton et al., 2015). Also, the resources and benefits brought by celebrity identity are far less than the advantages of state ownership. These findings shed light on the influencing mechanism and boundary conditions between celebrity CEOs and firm innovation strategy.

### Theoretical contributions

This study has several critical implications for management research and theory. First, by introducing identity theory into the research framework of upper echelon theory, this study analyzes the effect and underlying influence mechanisms of celebrity CEOs on firms’ innovation investment from a psychological perspective. It extends the research on the economic effects of CEOs’ celebrity status and the relationship between executives’ social characteristics and firm strategies. Specifically, it provides unique insights into executives’ decision-making motivation, managerial behavior, and differentiated corporate strategies in different social identities and status. We also examined the facilitating and constraining conditions that may affect the association between celebrity CEOs’ and innovation investment. Our study suggests that various organizational and environmental conditions can be key factors influencing CEOs’ celebrity effect, which in turn amplifies or inhibits celebrity CEOs’ motivation to engage in innovation strategy. By identifying the influencing mechanism and boundary conditions that celebrity CEOs’ effort in firm behaviors, we advance research on identity theory and upper echelon theory.

Second, we contribute to the innovation management literature by exploring the executives’ psychological factors on innovation practices and investment. Previous studies have mainly focused on the effect of executives’ demographic characteristics on innovation activities (Hambrick, 2007; Lin et al., 2011; Heyden et al., 2017). Recently, scholars have investigated how strategy decisions and activities are directly influenced by psychological traits, including overconfidence, narcissism, and hubris (Park et al., 2018; Tang et al., 2018; Li and Zhang, 2022; Wang et al., 2022). In line with these studies in the subfield of innovation management, we explored the impact of CEOs’ celebrity status on firm innovation investment by suggesting social status as a behavioral reference for decision-making. Our results revealed that the CEOs’ internalization process of celebrity status into their own identity and desire for preserving celebrity identity are significant determinants of firm innovation activities. It extends the research on the antecedents of innovation input, and contributes to the literature on the relationship between executives’ psychological traits or their individual social needs and firm innovation activities.

Third, our results may offer a potential solution for agency issues. They revealed that innovation activities of firms are not only affected by executives’ demographic characteristics, but also by the interaction between executives and society. Compared to demographic characteristics, social characteristics is more guidable and exploitable, which can be used to strengthen firms’ innovation activities. According to agency theory, the core problem of corporate governance is the contradiction between agents’ short-term personal interests and firms’ long-term profitability (Matta and Beamishi, 2008). Since innovation strategy tend to be high-risk, high-input, long-term, uncertain, and does not always result in the desired future performance, it can contribute to risk averseness on the part of CEOs and their selection of activities that lead to short-term returns (Lin et al., 2011). However, media reports and the desire to receive awards and become a business celebrity can establish intangible standards and expectations that are attached to executives with celebrity identity (Zavyalova et al., 2017). Such standards and expectations constitute in effect an external corporate governance mechanism outside the board of directors and the company, which can serve to discipline the behavior of executives through their desire to maintain their celebrity status. Such mechanism can restrain CEOs’ risk aversion preferences, make firms’ long-term interests consistent with the CEOs’ personal interests. The source of this external corporate governance restraint is the significance of the reputation and status of the CEO, which is monitored by the whole society relying on moral restraints. Thus, the discrepancy between the long-term interests of a firm and executive’s aversion to risk can be resolved through the mechanism of the executives’ celebrity status. We build a promising thought that resolves the principal-agent issue according to identity theory. This insight presents a possible method that could reconcile the varying interests of both firms’ stakeholders and CEOs.

### Practical implications

Our findings also provide practical implications. With the growing number of well-known business leaders frequently receiving media attention, celebrity CEOs can play a vital role in listed companies and society. This study reveals the economic significance in the relationship between celebrity CEOs and firms’ innovation strategies. It provides practical insights for boards of directors on making decisions about executives’ recruitment. The boards should ensure that the motivation of CEOs and their decisions are in accordance with the firms’ innovation strategy. To improve innovation capabilities and achieve a technical advantage, the directors should recruit a celebrity CEO or encourage their current CEO to get an award. However, when celebrity CEOs cannot meet the expectations of firms’ stakeholders and the society, they tend to realize that there is a possibility of losing their status and reputation. They may excessively engage in innovation without careful strategic considerations of costs and risks, which is more likely to damage firms’ interests. Moreover, the celebrity status, which can lead to CEOs’ overconfidence and narcissism, will further exacerbate this tendency. Thus, the board of directors should also monitor the CEOs’ risk-taking behavior to prevent any potential loss owing to the excessive risk-taking behaviors exhibited by celebrity CEOs to achieve personal goals.

### Limitations and future research

This study has the following limitations. First, we only employed R&D intensity to measure innovation input which also includes research staff, innovation platform, research cooperation etc. Our measurement can only partially represent firms’ innovation willingness and limits the research between celebrity CEOs and innovation. Future research should employ more variables to measure innovation input from various perspectives. Second, we did not investigate to what extent greater engagement in innovation investment by celebrity CEOs would actually benefit to maintain their status and reputation. The association between innovation input and CEOs’ celebrity status has not been built directly. Therefore, future studies should further explore how different degrees of increase in R&D expenditures positively affect celebrity CEOs’ social position or the likelihood of maintaining their celebrity status. Third, we did not consider the time effect of celebrity status. However, external praise of celebrity CEOs becomes less frequency and less important over time, making it difficult to provide sufficient motivation for identity control (Lovelace et al., 2018). A recently acquired celebrity status is more likely to have a significant impact on the CEO’s decision-making than an earlier one (Cialdini and Goldstein, 2004). The time effect may complicate the impact of celebrity CEOs’ on innovation input. In future research, it would be interesting to examine the effect of time on celebrity CEOs’ engagement in innovation investment.

## Data availability statement

The raw data supporting the conclusions of this article will be made available by the authors, without undue reservation.

## Author contributions

DS and KL contributed to the theoretical framework, writing and formatting of the research. KL and SZ contributed to the supervision and review. DS, SZ, and SW contributed to the editing and analysis of the research. All authors contributed to the article and approved the submitted version.

## Funding

This work was supported by the National Natural Science Foundation of China (grant number: 72174073).

## Conflict of interest

The authors declare that the research was conducted in the absence of any commercial or financial relationships that could be construed as a potential conflict of interest.

## Publisher’s note

All claims expressed in this article are solely those of the authors and do not necessarily represent those of their affiliated organizations, or those of the publisher, the editors and the reviewers. Any product that may be evaluated in this article, or claim that may be made by its manufacturer, is not guaranteed or endorsed by the publisher.
